# Examining the lineage autonomous role of β3‐integrin in muscle regeneration

**DOI:** 10.1096/fj.202200464

**Published:** 2022-06-23

**Authors:** Nathalie Gerassimov, Colt Crain, Colin Bilyeu, Andrew Jacob, Chen‐Ming Fan

**Affiliations:** ^1^ Department of Embryology Carnegie Institution for Science Baltimore Maryland USA; ^2^ Department of Cell, Molecular and Developmental Biology Johns Hopkins University Baltimore Maryland USA; ^3^ Seraxis Inc. Germantown Maryland USA

**Keywords:** differentiation, mouse, muscle stem cells, regeneration, β1‐integrin, β3‐integrin

## Abstract

Skeletal muscles can regenerate over the lifetime from resident muscle stem cells (MuSCs). Interactions between MuSCs and extracellular matrix (ECM) proteins are essential for muscle regeneration. The best‐known receptors for ECM proteins are integrins, a family composed of twenty‐some heterodimeric combinations of an α‐ and a β‐subunit. β1‐integrin (encoded by *Itgb1*) is required for quiescence, proliferation, migration, and fusion of Pax7^+^ MuSCs in the mouse model. β3‐integrin (encoded by *Itgb3*) has been reported to be critical for the myogenic differentiation of C2C12 myoblasts, and *Itgb3* germline mutant mice were shown to regenerate few if any myofibers after injury. To investigate the autonomous role of *Itgb3* in the myogenic lineage in vivo, we conditionally inactivated a floxed *Itgb3* allele (*Itgb3^F^*) by constitutive *Pax7*‐*Cre* and tamoxifen‐inducible *Pax7*‐*CreERT2* drivers. Unexpectedly, we found no defects in muscle regeneration in both conditional knockout models. In vitro studies using *Itgb3* mutant myoblasts or RNAi knockdown of *Itgb3* in myoblasts also did not reveal a role for myogenic differentiation. As β1‐ and β3‐integrins share ECM ligands and downstream signaling effectors, we further examined *Itgb3's* role in a *Itgb1* haploid background. Still, we found no evidence for an autonomous role of *Itgb3* in muscle regeneration in vivo. Thus, while *Itgb3* is critical for the differentiation of C2C12 cells, the regenerative defects reported for the *Itgb3* germline mutant are not due to its role in the MuSC. We conclude that if β3‐integrin does have a role in Pax7^+^ MuSCs, it is compensated by β1‐ and/or another β‐integrin(s).

**Abbreviations**
cKO: conditional knockout
DPI: days post injury
F: floxed
FACS: fluorescence‐activated cell sorting
iKO: inducible knockout
MuSCs: muscle stem cells
qRT‐PCR: quantitative reverse transcription‐polymerase chain reaction
RNAi: RNA interference

## 1 INTRODUCTION

Skeletal muscles are essential for locomotion. Research spanning many decades has provided us with a fundamental framework of their origin, formation, and regeneration, as well as pathology in aged and diseased conditions.^1^, ^2^ Lineage tracing in mice reveals that cells descending from dermomyotome of the somite, expressing *Pax3* and/or *Pax7*, give rise to the trunk and limb musculatures during embryonic development.^3^, ^4^ At late fetal stages, the *Pax3*‐expressing myogenic progenitor population diminishes,^3^ whereas the *Pax7*‐expressing progenitor population expands and contributes to the growth of postnatal muscles, as well as to the establishment of muscle stem cells (MuSCs) in adult mice.^5^ *Pax3*‐expressing MuSC populations do exist in select muscle groups in post‐natal mice.^3^, ^6^ Contrasting to cardiac muscles, skeletal muscles have a remarkable ability to regenerate throughout life, despite this ability waning over time.^7^, ^8^ With the exception of Type II myofibers,^9^ regenerated muscle fibers are mainly derived from *Pax7*‐expressing muscle stem cells (Pax7^+^ MuSCs).^5^ Indeed, ablation of *Pax7*‐expressing cells in adult mice leads to failed muscle regeneration after acute injury.^10^, ^11^, ^12^ For many recent studies cited above, the Pax3‐ and Pax7‐driven genetic tools have been invaluable for manipulation of gene expression and function in the myogenic lineage in mice, including studies of stem cell quiescence, activation, proliferation, self‐renewal, and differentiation.^7^, ^8^, ^13^

The role of the extracellular matrix (ECM) for skeletal muscle is of particular importance.^14^, ^15^ Mutations in genes encoding ECM proteins have been linked to muscular dystrophies. For examples, mutations in *LAMA2* (encoding laminin‐α2) cause congenital muscular dystrophy type IA to varying degrees,^16^, ^17^ and mutations in *COL6 (*encoding collagen type VI) cause Bethlem myopathy or Ullrich congenital muscular dystrophy, depending on the nature of each mutation.^18^ Mutations in genes encoding ECM receptors or ECM muscle tethering proteins also cause muscle dysfunction. For example, a mutation in *ITGA7* (encoding α7‐integrin, which with β1‐integrin forms a receptor for laminin) causes congenital muscular dystrophies.^19^, ^20^ The most frequently studied muscular disease is Duchenne's muscular dystrophy (DMD), which is caused by mutations in the *DMD* gene (encoding dystrophin).^1^, ^21^ Dystrophin tethers the actin cytoskeleton to α‐dystroglycan which is also a laminin receptor. In recent years, it has become clear that the ECM, ECM receptors, and their tethering proteins are expressed by MuSCs. Collagen V and VI have been implicated in MuSC quiescence and renewal,^22^, ^23^ while fibronectin and laminin have been implicated in MuSC maintenance and renewal.^24^, ^25^ Dystrophin regulates MuSC polarity and renewal,^26^ whereas β1‐integrin controls multiple aspects of MuSC function, from quiescence, proliferation, renewal, and migration to myocyte fusion.^27^ β1‐integrin is of particular interest as it can heterodimerize with many α‐integrins and form receptors for laminin, collagen, fibronectin, and a plethora of other ECM proteins present in the skeletal muscle.^14^, ^15^, ^28^ Intravital imaging of adult mouse muscles further supports the role of the ECM in guiding lineage‐marked *Pax7*‐descendent cells in early regeneration.^29^ Thus, interactions between MuSCs and the ECM deserve attention to further our understanding of regenerative muscle biology.

In addition to β1‐integrin, β3‐integrin (encoded by *Itgb3*) has been reported as critical for myogenic differentiation.^30^ Knocking down *Itgb3* by RNAi or inhibiting β3‐integrin by function‐blocking antibodies in C2C12 cells led to defective differentiation and fusion via a mechanism involving reduced activity of Rac1. Furthermore, *Itgb3* germline mutant mice had no regenerated myofibers at 3 days post injury (DPI). There was no report for a developmental defect of myogenesis in *Itgb3* germline mutant mice, suggesting that its role for myogenic differentiation is restricted to post‐natal muscle regeneration. The above findings are intriguing, in light of the fact that *Itgb1* cKO by HSA‐Cre causes defects in migration and fusion, but not differentiation, of myogenic cells during embryonic myogenesis.^31^ In adult mice, *Pax7*‐*CreERT2*‐driven, tamoxifen‐induced MuSC‐KO of *Itgb1* (*Itgb1*‐iKO) resulted in very few regenerated myofibers post‐injury, primarily due to a defect in stem cell proliferation.^27^ Furthermore, adult *Itgb1*‐iKO MuSCs showed accelerated expression of differentiation genes in vitro, suggesting that they are capable of differentiation.^27^ One may therefore infer that β3‐integrin has a distinct role from β1‐integrin. However, an earlier study by Blaschuk et al. (1997) in C2 myoblasts showed that β3‐integrin expression delayed, instead of promoted, myogenic differentiation and fusion. The differing strategies used to generate the *Itgb3* versus *Itgb1* mouse models in above studies also make direct inference problematic.

One main difference in deciphering the in vivo role of *Itgb3* versus *Itgb1* cited above is the use of an *Itgb3* germline mutant versus conditional mutants of *Itgb1*. Due to the early lethality of the *Itgb1* germline mutant,^19^, ^32^ an *Itgb1*‐floxed allele (*Itgb1^F^*; Ref. [33]) was inactivated by HSA‐Cre^31^ or Pax7‐CreERT2^27^ to study its autonomous function in the myogenic lineage, during embryonic and adult muscles, respectively. *Itgb3* germline mutant mice have been studied extensively since its original report.^34^ Among multiple documented roles, β3‐integrin is best known to dimerize with αIIb‐integrin and form the receptor on platelets for fibrinogen to produce blood clots.^35^ A prominent feature of *Itgb3* germline mutant mice is their premature death by 3 weeks of age due to severe hemorrhaging. One can therefore imagine that poor blood clotting at a muscle injury site could compromise regeneration, especially when assayed at three DPI when new myofibers are just about to form. Thus, one cannot be certain of its autonomous role in the myogenic lineage based on the germline mutant, per se. β1 and β3 are the two most studied β‐integrins, and they share binding substrates and intracellular signaling adapters.^36^, ^37^ Indeed, they have been shown to be functionally redundant in a breast cancer cell line select cell context.^38^ It has yet to be determined if there is a unique and noncompensatory role in the myogenic lineage for *Itgb3*. Given that a well‐characterized *Itgb3*‐floxed (*Itgb3^F^*) allele exists^39^; here, we examined its autonomous role in the *Pax7*‐lineage, using constitutive Pax7‐Cre (for cKO) and tamoxifen‐inducible Pax7‐CreERT2 (for iKO) drivers.

## 2 MATERIALS AND METHODS

### 2.1 Mouse models

*Pax7*‐*CreERT2*,^5^ *Pax7*‐*Cre*,^40^ *Itgb3^F^*,^39^ *Itgb1^F^*,^33^ and *R26R*‐*YFP* ^41^ alleles were published and characterized, and obtained either from original investigators or the Jackson Laboratory (JAX).

### 2.2 Tamoxifen regimen

Female and male mice between 3 and 6 months of age (as adult mice) were used. They were injected intraperitoneally daily with tamoxifen (tmx at 10 µl/gram of body weight) for five days. After a 7 day recovery period (for the clearance of tmx and recombination‐induced knockout), mice were used for experiments such as cell isolation or injury.

### 2.3 Tibialis anterior (TA) muscle Injury

Mice were anesthetized by isoflurane/oxygen vapor or Avertin (2.5% solution, 10 µl per gram body weight). Intramuscular injection of 50 μl of 2.5 μM cardiotoxin (EMD) using an insulin syringe (U‐100, Becton Dickinson) was used to induce injury. Animals were euthanized by cervical dislocation, and their TA muscles were harvested at 5 or 28 days post‐injury (DPI). Uninjured TA muscles from mice of the same age (littermates used whenever possible) were used for comparison. Immediately after dissection, TA muscles were fixed in ice‐cold 4% paraformaldehyde (PFA; EMS) in PBS for 8–10 min, followed by a 30‐min cold PBS wash. TA muscles were subjected to 10%, and 25% sucrose impregnation, followed by 2 h in 1:1 solution of 25% sucrose/OCT, vertically positioned in tragacanth gum pasted on corks for cryo‐embedded in OCT using isopentane (Sigma)/liquid nitrogen. Samples were stored in −80°C freezer until sectioning. The cross sections of the mid‐belly region of the TA were at 10 μm thickness and stored in −20°C freezer, until ready for histological staining by Gill's II hematoxylin and eosin (H&E; Surgipath) and immunostaining.

### 2.4 Immunostaining of muscle tissue sections

TA muscle sections were air‐dried at room temperature (RT) for 30 min, washed with phosphate‐buffered saline (PBS) to remove the OCT, permeabilized with 0.5% TritonX‐100/PBS for 20 min, and rinsed with 0.05% TritonX‐100/PBS (PBST) for 5 min. Slides were then incubated with Avidin at RT for 15 min, washed with PBST, incubated with Biotin at RT for 30 min, and washed again with PBST. Slides were then incubated overnight at 4°C with M.O.M. blocking agent from Vector Laboratories (1.25 ml PBST +1 drop of M.O.M. stock), rinsed with PBST, treated with blocking buffer (10% normal goat serum and 10× carbo‐free blocking solution, Vector Labs) for 2 h, before being treated with primary antibody to Pax7 (at 1:10 dilution; DSHB) in blocking solution (see Primary Antibodies for dilutions) at 4°C overnight. They were then washed with PBST and incubated with Alexa‐488 conjugated secondary antibody to mouse IgG1 (at 1:1000 dilution; Invitrogen) in blocking along with DAPI for 45 min at RT, washed with PBST, and mounted with Fluoromount‐G (Southern Biotech Inc) and coverslip for imaging.

### 2.5 FACS Isolation of MuSCs

MuSCs were isolated following the previously described protocol,^42^ with slight modifications. Briefly, skeletal muscles were dissected, minced, and incubated in 0.2% Collagenase Type I (Sigma) in Ham's F‐10 Nutrient Mix (F‐10; Gibco) at 37°C with gentle shaking for 1.5 h followed by centrifugation and wash. Digested tissues were then incubated in 0.2% Dispase (Gibco) in F‐10 and Collagenase at 37°C with gentle shaking for 0.5 h. Cell suspension was then subjected to mechanical dissociation using a syringe and a 20‐gauge needle and filtered through a 40‐μm cell strainer (VWR). Next, the cell suspension was filtered through a 35‐μm cell strainer and incubated with DAPI for 5 min.  Cells were then subjected to fluorescence‐activated cell sorting (FACS) using the ARIA III sorter (BD Biosciences) and data were collected by BD FACSDiva software. YFP‐positive and DAPI‐negative single cells were collected in FACS wash medium (10% HS in F‐10 medium).

### 2.6 Primary myoblast culture

Prior to cell seeding, culturing dishes (12, 24, and 48 wells) were coated with Matrigel (Corning, 354248; 30 min at 37°C or overnight at 4°C) and filled with MuSC growth medium: 10 ng/ml FGF, 20% fetal bovine serum (FBS), 5% HS, 1% Pen/Strep, 1% Glutamax (Gibco), 0.1% chick embryo extract (MP biomedicals) in DMEM (Gibco). YFP‐positive and DAPI‐negative cells were plated at appropriate cell densities into the culturing plates and cultured at 37°C in tissue culture incubators with 5% CO_2_ for 4 days to allow for cell expansion and downstream assays.

### 2.7 Differentiation and fusion assays

For assaying of differentiation and cell–cell fusion, myoblasts were switched to differentiation medium (2% HS, 1% Pen/Strep in DMEM). Each well was washed twice with 500 µl of DMEM per well without serum before switching to 500 µl of differentiation media. Differentiation media were changed daily. After 3 days, cells were fixed at day 3 in 4% PFA (in PBS) for 5 min, and processed for immunostaining with monoclonal antibody supernatants to MyoG (at 1:40 dilution, F5D) and myosin heavy chain (at 1:50 dilution, MF 20) from DSHB, followed by secondary antibodies to each isotype (Alex 568 anti‐IgG1 and Alex 488 anti‐IgG2b, 1:1000 each; Invitrogen), and DAPI staining. The differentiation index was calculated as the percentage of nuclei‐expressing MyoG. The fusion index was calculated as the percentage of nuclei within the MF 20‐expressing myotubes; only those with at least two nuclei in a contiguous MF 20+ domain were included.

### 2.8 Western blot analysis

Western blot analysis was performed using cell lysates prepared in T‐PER tissue protein extraction reagent (Thermo) with protease and phosphatase inhibitor cocktail (Roch). After resolved by SDS‐PAGE (4%–20% TGX gradient gel, BioRad), proteins were transferred to PVDF membrane (Bio‐Rad), rinsed in PBST, blocked in 5% dry milk, and incubated with a rabbit monoclonal antibody against mouse β3‐Integrin (at 1:1000 dilution, #13166; Cell Signaling Technology) and a mouse monoclonal antibody for GAPDH (at 1:1000 dilution, #RDI‐TRK5G4‐6C5; Research Diagnostics Inc). HRP‐conjugated goat anti‐rabbit secondary antibody (1:3000, Bio‐Rad) and goat anti‐mouse secondary antibody (1:3000, Invitrogen) were used for detection by chemiluminescence (SuperSignal WestPico PLUS; Thermo). Signals were obtained by an LICOR‐Odyssey scanner.

### 2.9 RNAi

Primary myoblasts isolated from 3‐month‐old C57/Bl6 males were differentially plated for enrichment. At passages 3–4, they were seeded at 25% confluency in 500 µl of growth media in 24‐well dishes coated with Matrigel. For each well, 100‐µl transfection mixture containing Lipofectamine RNAiMax (2 µl in 50 µl OPTI‐MEMI)(Invitrogen) and duplex stealth oligos (30 pmol of in 50 µl OPTI‐MEM) was added immediately after cell seeding. For blank controls, 100 µl of OPTI‐MEM was added at the same time. Two days after transfection, myoblasts were harvested for qRT‐PCR, or subjected to differentiation for 3 days.

### 2.10 qRT‐PCR

RNA samples were harvested by Trizol (Invitrogen). For each well of cultured myoblasts (in 24‐well) dish, 300 µl of Trizol per well was used, followed by precipitation procedure in the manufacturer's manual. Glycogen blue (Ambion) was spiked in to aid visualization of the pellet. Each RNA pellet was resuspended in 20 µl of water and stored at −80 for subsequent use. For reverse transcription (RT), 1 µl of each RNA sample was used for cDNA synthesis in a 20‐µl reaction using the QuantiTect RT kit following manufacturer's protocol (Qiagen). For qPCR, 1 µl from each cDNA sample was used, together with oligo primer pairs (at 5 pmol each) to *Gapdh* and *Itgb3* and Sybergreen 2X mix (BioRad) in a 14‐µl reaction for PCR using the Bio‐Rad CFX‐96 real‐time PCR machine (58°C annealing and for 35 cycles). Primers used are: *Gapdh*, 5′‐GCT CAT GAC CAC AGT CCA TGC and 5′‐GGA TGC AGG ATG TTC TGG (product = 111 bp); *Itgb3*, 5′‐AGC AGC GAC TTC GGC AAG ATC AC and 5′‐CAT TGG TGG ACA TGC AGG TGT CA (products = 187 bp). The delta‐delta *C* values (i.e., normalized to *Gapdh*) were calculated and normalized to blank controls for changes in relative expression levels.

### 2.11 Image acquisition and quantification

The H&E‐stained TA muscle sections were imaged with a Nikon 800 microscope using 20× Plan Apo objectives and Canon EOS T3 camera using EOS Utility acquisition software. Fluorescent imaging of TA muscle sections was captured using a Nikon Eclipse E800 microscope with 20×/0.50 Plan Fluor oil objectives and Hamamatsu digital camera C11440 using MetaMorph Microscopy Automation and Image Analysis Software. For fluorescent imaging of myoblasts in vitro, images were captured using a Nikon Ti2 inverted microscope controlled by the Element software with 20×/0.50 Plan Fluor long distance objective. For quantification of muscle fiber number, diameter, cell density, and differentiation/fusion indices, images were analyzed in ImageJ (v 1.44p, NIH, Bethesda), an image processing program. Pixel to μm ratios were determined by imaging a micrometer using the same settings for each set of data, and used to size the scale bars.

### 2.12 Statistical analysis

Summary statistics (mean and standard deviation) describing the minimum ferret diameter and density of muscle fibers in sections from individual mice were combined into experimental and control summary statistics via Cochrane's formula. The statistical significance of results was determined by one‐tailed unpaired Student's *t*‐test when comparing control and experimental groups, and by two‐tailed unpaired Student's *t*‐test when comparing genotypes, both with an *ɑ* of 0.05. Distributions of fiber diameter between experimental and control groups were compared via the Kolmogorov–Smirnov test with an *ɑ* of 0.05 for all.

## 3 RESULTS

### 3.1 *Pax7*‐*Cre*‐mediated *Itgb3* mutant mice develop normal skeletal muscles

**FIGURE 1:**
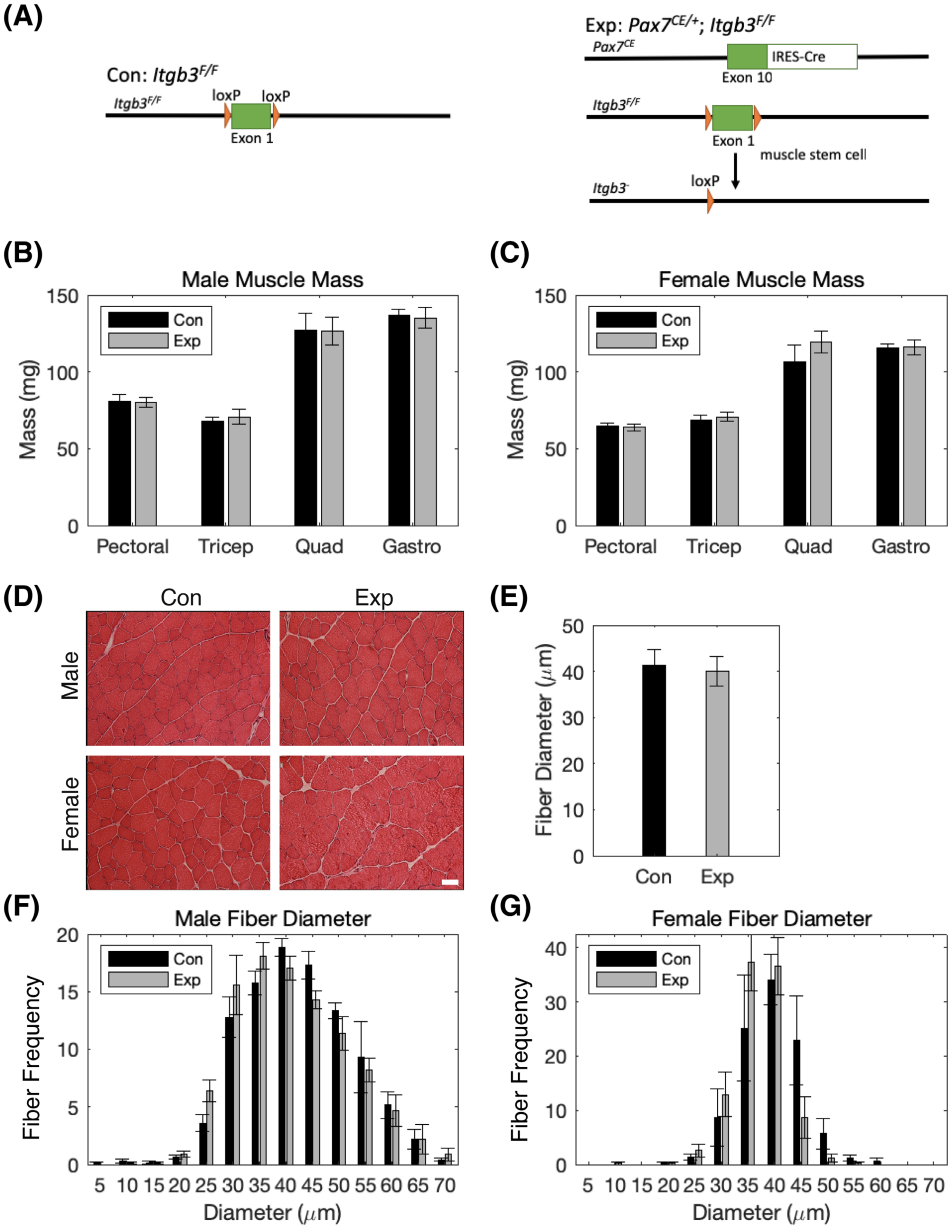
*Pax7*‐*Cre*‐mediated *Itgb3* cKO mice develop skeletal muscles normally. (A) Genetics of control (Con, *Itgb3^F^* ^/^ *^F^*) and experimental (Exp: *Pax7^CE^* ^/+^;*Itgb3^F^* ^/^ *^F^*) mice. The *Itgb3* conditional knockout (cKO) mice have the floxed *Itgb3* Exon 1 excised by the Cre‐recombinase expressed from the Pax7 locus (the IRES‐Cre cassette is inserted in the last exon). (B and C) Mass of pectoral, tricep, quadricep (Quad), and gastrocnemius (Gastro) muscles of control and experimental mice for males (B) and females (C) at 3 months of age. *N* = 5 mice per sex, for each control and experimental group. (D) Histological photomicrographs of sectioned tibialis anterior (TA) muscles of control and experimental groups at 3 months of age. *N* = 5 mice per sex for each control and experimental group. Scale bar, 50 µm. (E) Averaged (minimal ferret) diameter of myofibers for control and experimental groups based on data in (D); males and females combined. (F and G) Distributions of myofiber diameters of TA muscle fibers in males (F) and females (G) of control and experimental mice, using data from (E). For (F and G), the control and experimental distributions were subjected the Kolmogorov–Smirnov test, and found not to significantly different with *D* = .0768 for (F) and *D* = 0.1657 for (G), both <(*D*_Critical, *ɑ*=0.05_ = 0.624). In bar graphs, control groups are represented by black bars; experimental groups, gray bars; and error bars are the standard error of mean. Data were subjected to Student's *t*‐test. No significant differences (*p* > .05) were found, hence not indicated.

To achieve muscle lineage‐specific inactivation of *Itgb3*, we generated mice homozygous for an *Itgb3^F^ allele (Itgb3^F^* ^/^ *^F^*),^39^ in the absence or presence of a *Pax7*‐*Cre* allele,^40^ referred to as the control and *Itgb3* cKO experimental mice, respectively (Figure 1A). *Itgb3* cKO mice live to adulthood, are fertile, and bear no obvious difference in appearance (up to 1 year) to their littermate controls.

If *Itgb3* functions in muscle differentiation, the cKO mice would have smaller muscles than controls. Thus, we measured the weight of multiple muscle groups, including the pectoral, triceps, quadricep, and gastrocnemius muscle of 3‐month‐old male and female mice (Figure 1B,C, respectively). In either sex, we did not find a significant difference in these muscle groups between cKOs and controls. We then performed histological examination of samples prepared from their tibialis anterior (TA) muscles (Figure 1D). Using these histological data, we quantified myofiber diameters of control and experimental samples, but did not find a significant difference between them by averaged diameters (sex combined; Figure 1E). When we separated male and female samples and plotted the distribution pattern of myofiber diameters in each sex, we also did not find significant differences between control and experimental samples in either gender (Figure 1F,G). Thus, inactivation of *Itgb3* by the *Pax7*‐*Cre* driver does not reveal an autonomous role in muscle development and maturation.

### 3.2 *Pax7*‐*Cre*‐mediated *Itgb3* mutant mice regenerate muscles normally

**FIGURE 2:**
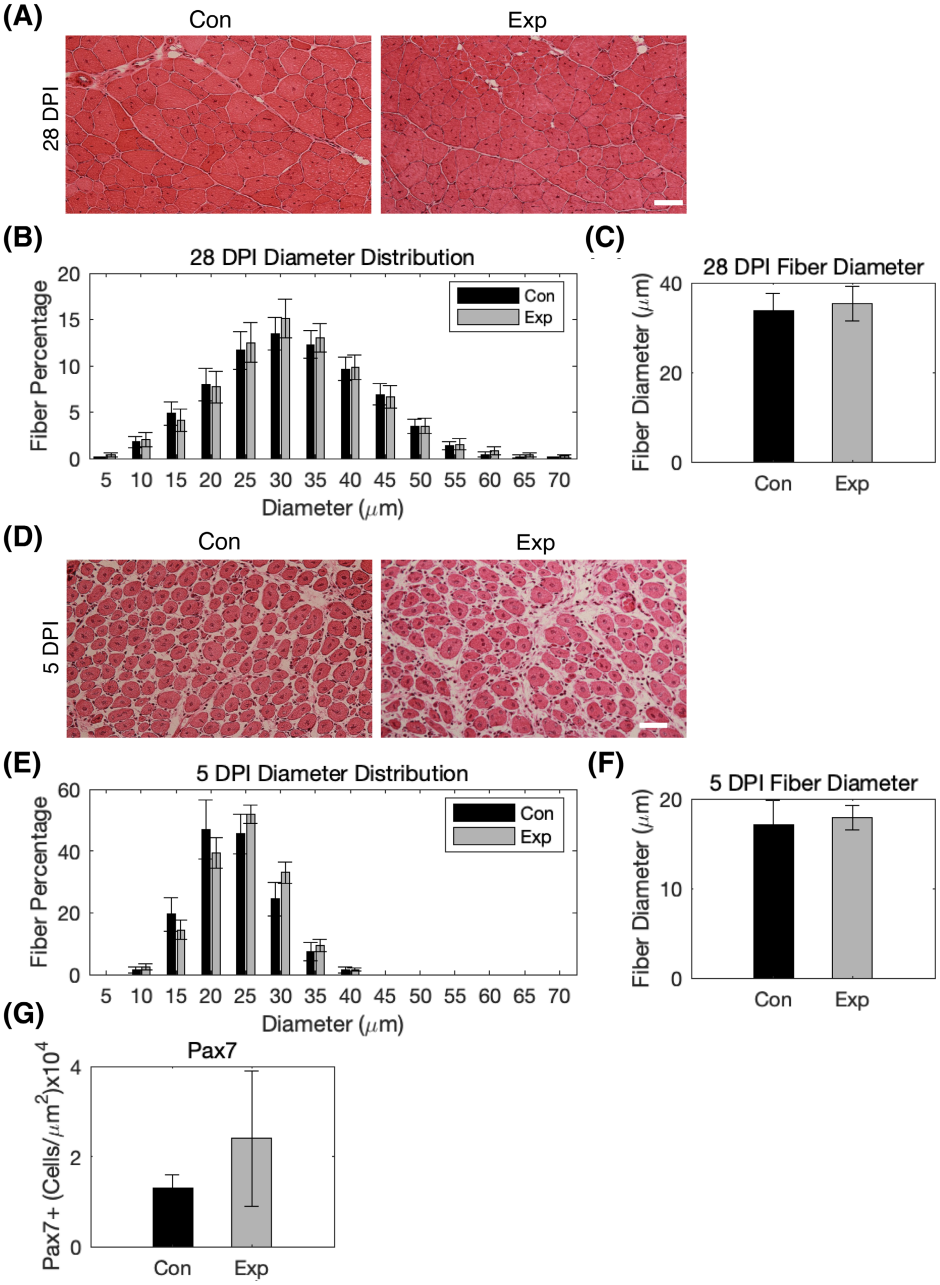
*Pax7*‐*Cre*‐mediated *Itgb3* mutant mice regenerate muscles normally. (A) Histological photomicrographs of control and experimental TA muscle fibers at 28 DPI; genotypes same as in Figure 1. Scale bar, 50 µm. Regenerated myofibers were identified by their centrally located nuclei. (B) Distributions of myofiber (minimal ferret) diameters in control (black bars) and experimental (gray bars) mice at 28 DPI. *N* = 8 per control and experimental group, male and female combined. The control and experimental distributions were compared via the Kolmogorov–Smirnov test and were found not to be significantly different with (*D* = 0.1497) < (*D*_Critical, *ɑ*=0.05_ = 0.454). (C) Averaged myofiber diameters derived from data in (C). (D) Histological micrographs of TA muscle sections at 5 DPI. (E) Distributions of muscle fiber diameters in control (black) and experimental (gray) mice at 5 DPI. *N* = 8 per control and experimental group, male and female combined. The control and experimental distributions were compared via the Kolmogorov–Smirnov test and were found not to be significantly different with *D* = 0.1307 (<*D*_Critical, *ɑ*=0.05_ = 0.454). (F) Averaged myofiber diameters derived from data in (E). (G) Quantification of Pax7^+^ cell density in uninjured control and experimental TA muscle sections. In bar graphs, control groups are represented by black bars, experimental groups by gray bars, and error bars are the standard error of mean. No significant differences (*p* > .05) were found between control and experimental group data by Student's *t*‐test, hence not indicated.

We next asked if *Itgb3* plays a role in muscle regeneration in adult mice when it is inactivated in the myogenic lineage. To test this, we performed cardiotoxin (ctx)‐induced injury to the TA muscle as used in the *Itgb3* germline mutant study,^30^ and assessed the regenerative phenotype histologically. To our surprise, we found abundant regenerative muscle fibers (those with centrally located nuclei) in both control and experimental muscles at 28 days post‐injury (DPI; Figure 2A). Furthermore, the diameters of regenerative myofibers in cKOs and controls were not different, either compared between individual sexes or compared between sex combined data (Figure 2B for distribution pattern and Figure 2C for average).

It is possible that an early myogenic differentiation defect was present but was masked during the long regeneration period. To address this, we performed injury regeneration analyses at 5 DPI (Figure 2D). Again, we found no significant differences between control and experimental samples in their regenerated myofiber diameter distribution pattern or averaged diameters (sex combined, Figure 2E,F). One possibility is that *Itgb3* cKO mice have super numeral MuSCs which generate more progenitors and thus result in a normal regenerative appearance—even if their differentiation and fusion efficiency are lagging behind. For this, we quantified the Pax7‐positive (Pax7^+^) MuSC cells. We found no significant differences of Pax7^+^ MuSC density between cKOs and controls (Figure 2G). We therefore conclude that *Itgb3* does not show an autonomous in vivo role in the myogenic lineage for adult muscle regeneration.

### 3.3 *Pax7*‐*Cre*‐mediated *Itgb3* mutant myoblasts differentiate normally in vitro

**FIGURE 3:**
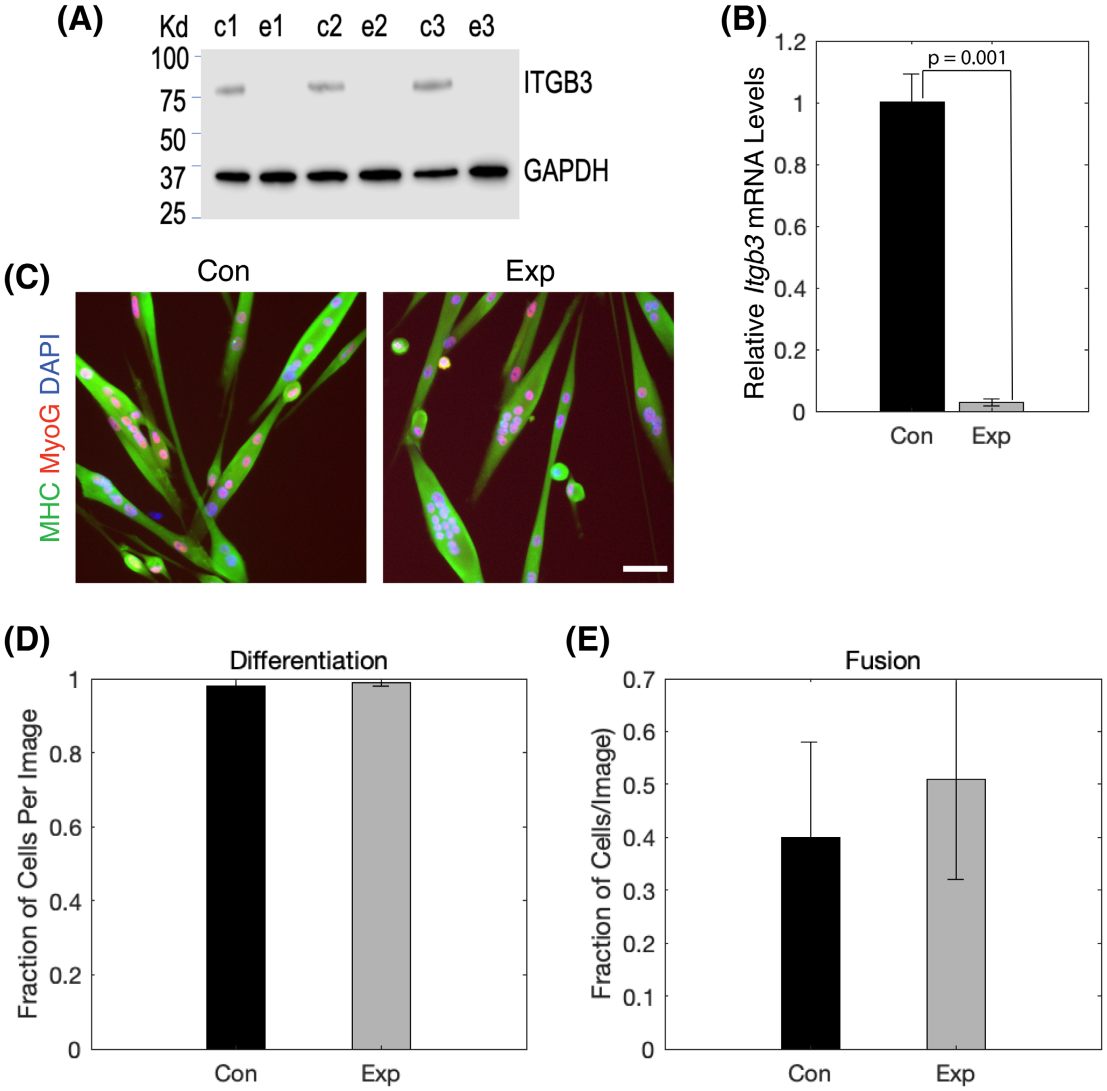
*Pax7*‐*Cre*‐mediated *Itgb3* mutant myoblasts differentiate normally in vitro. (A) Western blot of lysates prepared from myoblasts of three control (c1‐3) and three experimental (e1‐3) mice and probed for β3‐integrin; GAPDH was probed as a loading control. (B) Relative expression of *Itgb3* mRNA of control (scaled as 1) and experimental myoblasts by qRT‐PCR (normalized to *Gapdh* expression); *N* = 3 per for mouse gene Gapdh, instead of GAPDH.group, *p* = .001, by Student's *t*‐test. (C) Immunofluorescent images of control and experimental myoblasts subjected to differentiation media for 3 days, probed for MHC (green) and MyoG (red), and counterstained by DAPI (blue). *N* = 3 mice for each group. Scale bar, 50 µm. (D and E) Quantification of differentiation (D) and fusion (E) indices using data from (C). Myoblasts derived from each mouse were plated into three wells, and four to five images per well were taken for quantification. In bar graphs, control groups are represented by black bars, experimental groups by gray bars, and error bars are the standard error of mean. Student's *t*‐test was used for comparisons in (D) and (E), and no significant differences (*p* > .05) were found, hence not indicated.

The above data left us to wonder whether the *Pax7*‐*Cre* driver was ineffective in recombining the loxP sites at the *Itgb3* locus. Western blot analyses using extracts prepared from primary myoblasts revealed that there was little detectable β3‐integrin level in cKO myoblasts (Figure 3A). We also compared the levels of *Itgb3* mRNA between cKO and control myoblasts using quantitative reverse transcription polymerase reaction (qRT‐PCR). For this, a *R26^YFP^* Cre reporter allele^41^ was crossed in and used for cell marking and isolation by fluorescence‐activated cell sorting (FACS). YFP‐marked Pax7^+^ cells were purified from myoblasts prepared from *Itgb3* cKO and *Pax7*‐*Cre*;*R^YFP^* (control) hind limb muscles. We found a drastic reduction of *Itgb3* mRNA in the cKO (Figure 3B). These data support that β3‐integrin protein and *Itgb3* mRNA are effectively removed from the Pax7‐derived myogenic lineage by the Pax7‐Cre driver.

*Itgb3* knockdown by RNAi was shown to cause a defect in differentiation and fusion in C2C12.^30^ To examine if an acute knockdown of *Itgb3* would phenocopy in MuSC, we expanded MuSC‐derived YFP‐marked *Itgb3* mutant and control myoblasts in culture. Both populations displayed comparable cell growth. Next, we examined the effect of *Itgb3* RNAi on three independently prepared control and *Itgb3* cKO myoblasts (from three animals of each group) under differentiation inducing conditions (low serum media for 3 days). Differentiation and fusion were assayed by immunostaining for the differentiation markers myogenin (MyoG) and myosin heavy chain (MHC) using F5D and MF‐20 monoclonal antibodies, respectively, and quantified by counting % MyoG positivity and the % of nuclei in multinucleated MHC‐stained syncytia (Figure 3C). Unlike reported in C2C12, there was no significant differences in differentiation (Figure 3D) and fusion (Figure 3E) indices between control and *Itgb3* knockdown myoblasts.

### 3.4 Pax7‐CreER^T2^‐mediated inducible *Itgb3* mutants regenerate muscle normally

**FIGURE 4:**
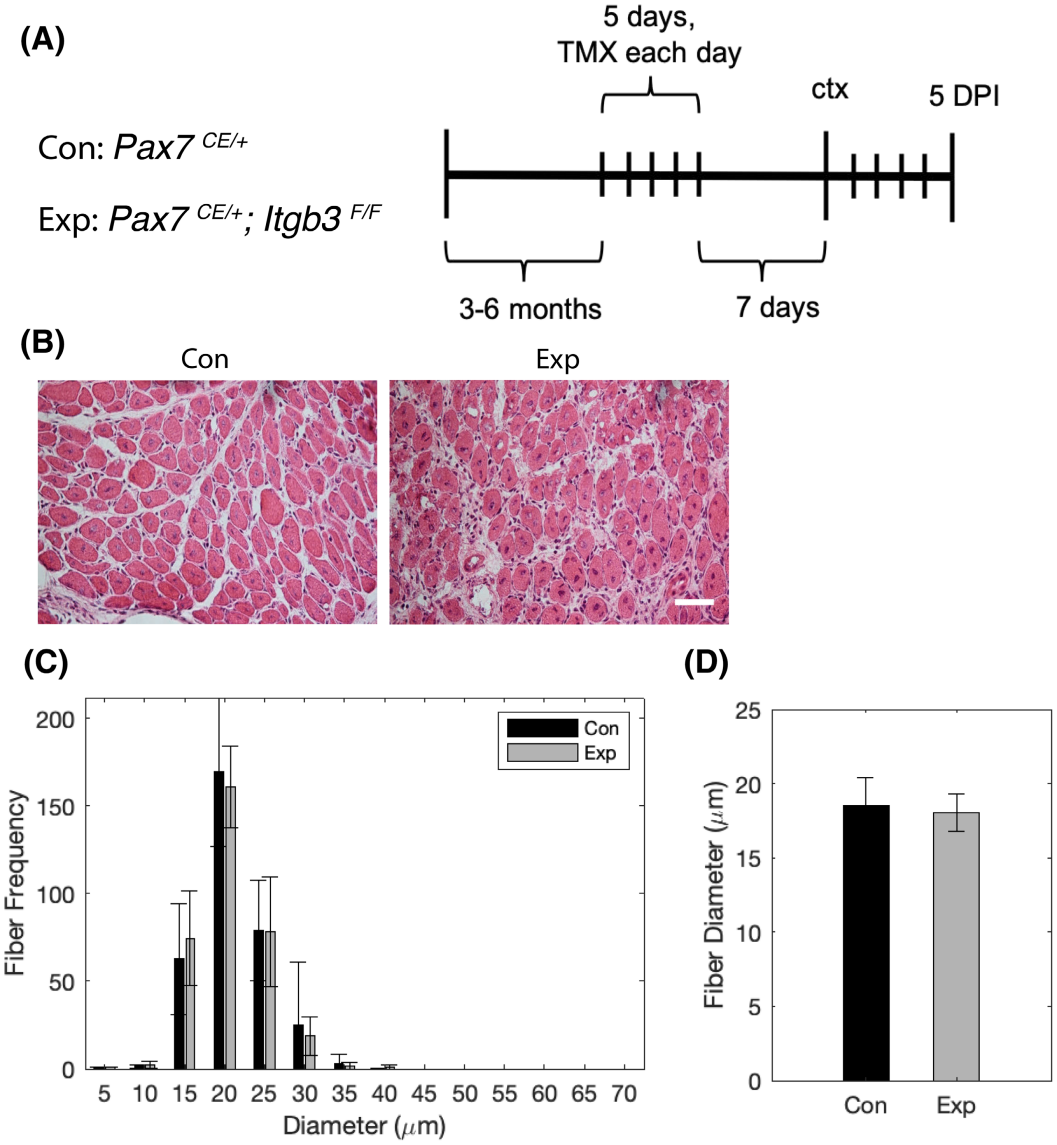
Pax7‐CreER^T2^‐mediated inducible *Itgb3* mutants regenerate muscle normally. (A) Control and experimental mouse genotypes (left) and experimental scheme (right). Mice of 3–6 months were injected daily with tamoxifen for 5 days, followed by a 7‐day washout period. The *Itgb3^F^* allele was inactivated after tamoxifen (TMX)‐induced Cre‐mediated recombination, that is, *Itgb3* iKO, the experimental group. Control mice did not harbor the *Itgb3^F^* allele. Their TA muscles were then injured by cardiotoxin (ctx) and harvested at 5 DPI. (B) Histological photomicrographs of control and experimental samples at 5 DPI. Scale bar, 50 µm. (C) The distribution of fiber diameters of regenerated fiber using data from (B), male and female combined. The control and experimental distributions were compared via the Kolmogorov–Smirnov test and were found not to be significantly different with *D* = 0.3472 (<*D*_Critical, *ɑ*=0.05_ = 0.519). (D) Averaged fiber diameters using data from (C). In (C and D), control groups are represented by black bars and experimental groups by gray bars, and error bars the standard error of mean. Student's *t*‐tests were used for comparisons, and no significant differences (*p* > .05) were found, hence not indicated.

We then considered the possibility that *Itgb3* cKO by *Pax7*‐*Cre* allows the myogenic lineage sufficient time throughout development to activate an unknown alternative mechanism for compensation. If so, acute inactivation of *Itgb3* in adult mice may uncover its role in regenerative differentiation. For this, we turned to using a *Pax7*‐*CreERT2* driver^5^ for tamoxifen‐induced *Itgb3* gene inactivation in adult mice (Figure 4A). We elected to concentrate our assessment of the *Itgb3* inducible KO (referred to as *Itgb3* iKO) phenotype on an early regeneration time point, 5 DPI (Figure 4B). However, neither fiber diameter distribution patterns (Figure 4C) nor averaged myofiber diameters (Figure 4D) at 5 DPI indicate significant differences between *Itgb3* iKOs and controls (sex combined).

### 3.5 dsRNA‐mediated *Itgb3* knockdown in primary myoblasts does not cause differentiation defects

**FIGURE 5:**
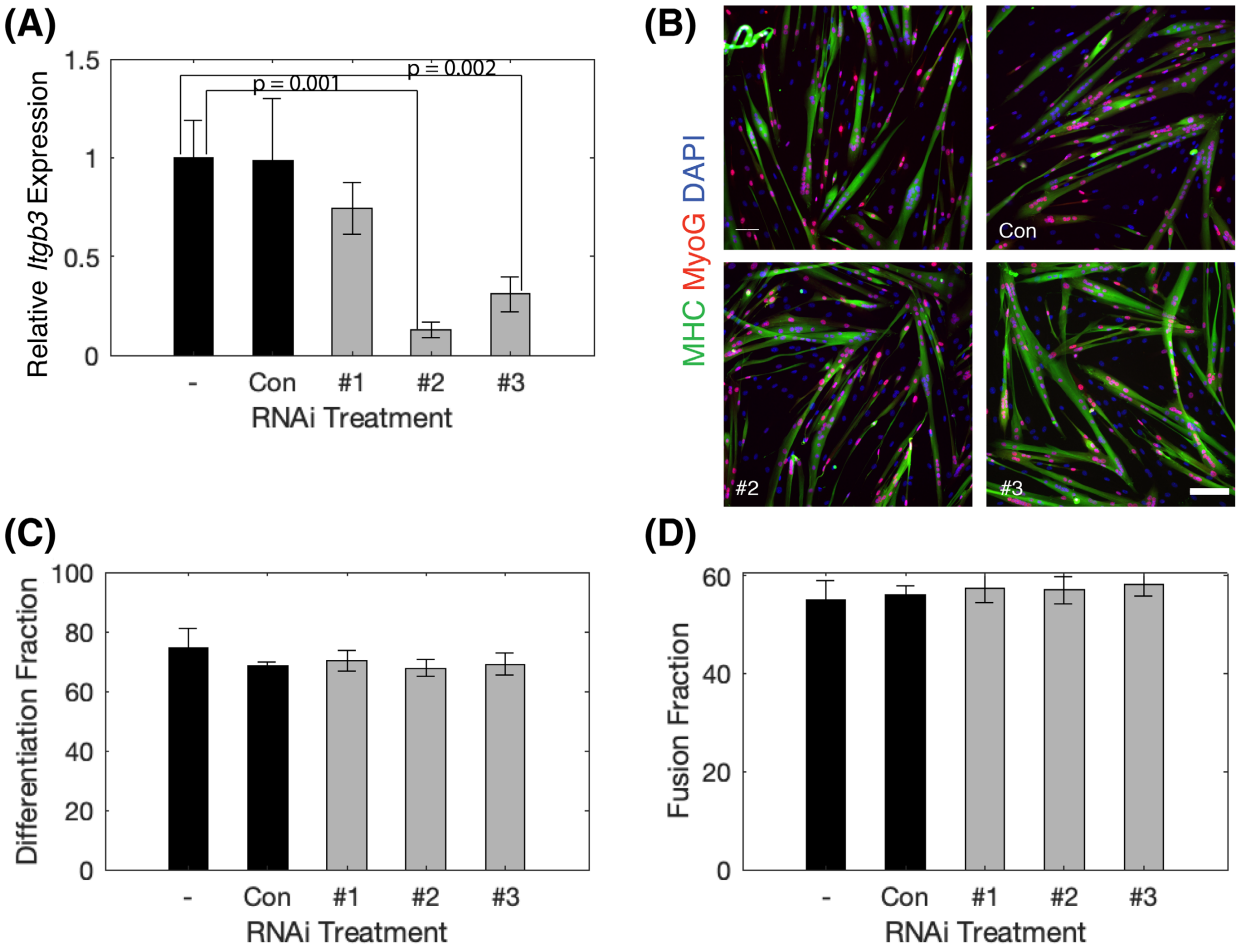
dsRNA‐mediated *Itgb3* knockdown in primary myoblasts does not cause differentiation defects. (A) Relative expression of *Itgb3* mRNA by qRT‐PCR to assess knockdown efficiencies of RNAi treatment by three independent shRNAs (#1–#3) in wild‐type myoblasts; “‐,” transfection without shRNA, and Con, with control shRNA. Expression levels are normalized to *GAPDH* and the data of “–” are scaled to 1. *N* = 3 for each treatment. Error bars are the standard error of mean. Statistical comparisons were performed pair‐wise to the “–” group for each treatment. Only those with *p* < .05 by Student's *t*‐test are labeled. (B) Fluorescent images of “–,” control, and shRNA‐transfected myoblasts differentiated for 3 days and probed for MHC (red) and MyoG (green), and counterstained by DAPI (blue); image of shRNA #1 is omitted for its low knockdown efficiency. Scale bar, 50 µm. (C and D) Differentiation and fusion indices, respectively, based on data in (B). *N* = 3 per RNAi treatment. Four images per well were taken for quantification. Student's *t*‐tests were used for statistical comparisons in (C) and (D), and no significant differences (*p* > .05) were found, hence not indicated.

We further considered the possibility that immediate RNAi knockdown of *Itgb3*, as opposed to 5 days of tamoxifen‐induced gene inactivation followed by a 7‐day washout period, was needed to reveal its role for myogenic differentiation prior to injury in vivo. Parallel to the prior C2C12 study, we prepared primary myoblasts isolated from C57/BI6 mice for RNAi. Three commercially available double‐stranded (ds) RNAs targeting *Itgb3*, siRNA #1‐3, were tested. As determined by qRT‐PCR (Figure 5A), dsRNA #1 had little or no effect to reduce *Itgb3* mRNA level compared to transfection with control dsRNA (Invitrogen) or no dsRNA (“–” in Figure 5A). dsRNAs #2 and #3 achieved knockdown efficiency at ~90% and 60%, respectively. Transfected myoblasts were then subjected to low‐serum induced differentiation for 3 days and assessed for differentiation and fusion (Figure 5B). We found no discernible difference in differentiation (Figure 5C) or fusion (Figure 5D) indices among mock and all dsRNA transfected groups.

### 3.6 The haploid insufficient phenotype of *Itgb1* is not exacerbated by *Itgb3* inactivation

**FIGURE 6:**
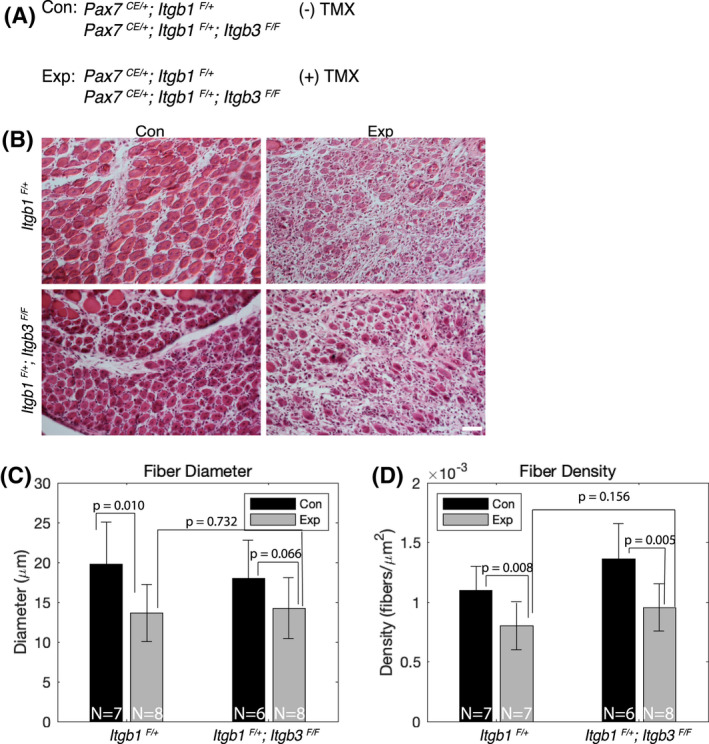
Haploid insufficient phenotype of *Itgb1* is not exacerbated by *Itgb3* inactivation. (A) Genotypes utilized for the following subfigures. Experimental (Exp) mice were treated with tamoxifen (+TMX), while control (Con) mice were not (−TMX). (B) Example photomicrographs of muscle fibers at 5 DPI for *Itgb1^F^* ^/+^ and *Itgb1^F^* ^/+^;*Itgb3^F^* ^/^ *^F^* control and experimental groups. The fiber size and density are notably different between control and experimental groups for each genotype. All images are of the same magnification. Scale bar, 50 µm. (C) Average (minimal ferret) diameters of muscle fibers at 5 DPI for control (black) and experimental (gray) groups for *Itgb1^F^* ^/+^ and *Itgb1^F^* ^/+^;*Itgb3^F^* ^/^ *^F^* genotypes. *p*‐Values for pair‐wised comparisons are indicated. There is a significant difference between the control and experimental *Itgb1^F^* ^/+^ genotype (*p* = .01), while there is a nonsignificant but discernible difference (*p* = .066) between the control and experimental *Itgb1^F^* ^/+^;*Itgb3^F^* ^/^ *^F^* genotype. (D) Average fiber density of muscle fibers at 5 DPI for control and experimental groups of *Itgb1^F^* ^/+^ and *Itgb1^F^* ^/+^;*Itgb3^F^* ^/^ *^F^* genotypes. *p*‐Values for each pairwised comparison are indicated: There are significant differences between the control and experimental groups within each *Itgb1^F^* ^/+^ and *Itgb1^F^* ^/+^;*Itgb3^F^* ^/^ *^F^* genotype, but not between the two experimental groups. For (C) and (D), *N* numbers for each group and genotype are indicated, males and females combined. *p*‐Values were determined by unpaired Student's *t*‐tests, with an *ɑ* of 0.05.

Finally, we entertained a possibility that *Itgb1* compensates for *Itgb3*, given that they share ECM ligands (e.g., fibronectin) and intracellular signaling components (e.g., integrin linked kinase).^36^, ^37^, ^43^ Because *Itgb1* iKO mice had virtually no regenerated myofibers,^27^ it was impossible to assess the genetic contribution of *Itgb3* in the *Itgb1* iKO background, to muscle regeneration. It was however possible to test *Itgb3* function in a background in which the *Itgb1* gene dosage was halved. For this, we generated *Itgb1^F^* ^/+^ and *Itgb1^F^* ^/+^ *Itgb3^F^* ^/^ *^F^* mice, in combination with *Pax7*‐*CreERT2* for tamoxifen‐induced gene inactivation (i.e., iKO). Mice of the same genetic combinations without tamoxifen injection were used for controls (Figure 6A). These mice were then subjected to injury and assessed at 5 DPI. Histological examination of control and experimental samples revealed that both *Itgb1^F^* ^/+^ iKO and *Itgb1^F^* ^/+^ *Itgb3^F^* ^/^ *^F^* iKO mice showed compromised regeneration (Figure 6B). Their fiber diameters were smaller than each of their respective controls (Figure 6C). Increased fibrotic area between regenerated myofibers was noticeable, resulting in a lower myofiber density per injury area (Figure 6D) in both iKO models. We do note that the fiber diameter difference between *Itgb1^F^* ^/+^ *Itgb3^F^* ^/^ *^F^* iKO and the corresponding control had a *p* value of .066 by *t*‐test, between .1 and .05. Importantly, the *Itgb1* ^+/−^ iKO data indicated a haploid insufficient phenotype in muscle regeneration. Thus, the level of *Itgb1* in MuSC is critical for regenerative capacity, extending our prior findings regarding the essential role of *Itgb1* in MuSC proliferation and renewal.^27^ However, *Itgb1^F^* ^/+^ *Itgb3^F^* ^/F^ iKO mice did not show a comparatively worsened phenotype either measured by myofiber diameter or density (Figure 6C,D). In both models, we did find many, albeit smaller, differentiated new myofibers (myofibers with centrally located nuclei), indicating that they are not completely blocked from differentiation. We thus showed no clear in vivo function of *Itgb3* in muscle regeneration even in an *Itgb1* haploid insufficient background, which argues against that the lack of *Itgb3* mutant phenotype in muscle regeneration is masked by an *Itgb1* compensation.

## 4 DISCUSSION

The role of integrins and their ECM ligands in tissue morphogenesis is well documented in many biological contexts. Their role in regulating MuSCs and muscle regeneration has recently come into light. Particularly relevant here is the discovery of the critical role of β1‐integrin for adult MuSC quiescence and self‐renewal by the study using Pax7‐CreERT2‐driven *Itgb1* iKO.^27^ The *Itgb3* germline mutant was reported to have little to no regenerative myofibers 3 days after injury,^30^ but myogenic lineage‐specific inactivation of *Itgb3* was lacking. Here, we evaluated the autonomous role of *Itgb3*, using cKO and iKO of *Itgb3* in the Pax7^+^ myogenic lineage in mice. These mouse models allowed us to better assess the relative contribution of *Itgb1* versus *Itgb3*. Despite our best efforts and multiple approaches, we were unable to document a clear role of *Itgb3* for regenerative myogenesis, either alone or in the *Itgb1* haploid‐insufficient background. The *Itgb3^F^* allele used herein deletes the promoter and first exon of *Itgb3* after Cre‐mediated recombination.^39^ Consistent with the original publication, we detected little protein and transcripts (using primers across the last two exons) in cKO myoblasts, thus excluding inefficient gene inactivation or hypomorphic allele as an explanation for the failure of documenting a role for *Itgb3*. We propose the following explanations for reconciliation. Most *Itgb3* germline mutants died from hemorrhaging and the survivors to adulthood still suffer this infliction.^34^ The muscle regeneration defects reported in this model^30^ may be secondary due to blood clotting defects. It is also possible that non‐myogenic cells expressing *Itgb3* are critical to support muscle regeneration. Either way, our data indicate that deficient muscle regeneration observed in the *Itgb3* germline mutant is not due to its function in the myogenic lineage.

While the C2C12 myoblast line is a powerful cell model for myogenesis, we opted to use primary myoblasts for their in vivo relevance. Yet, we were not able to document a role for *Itgb3* in myogenic differentiation using *Itgb3*‐deficient or *Itgb3*‐RNAi knockdown myoblasts in vitro, contrasting that which was reported for C2C12 myoblasts.^30^ Intriguingly, a prior study examined the dynamic changes of β3‐integrin during myogenic differentiation of C2 myoblasts and concluded that reducing, instead of increasing, β3‐integrin was critical for differentiation to take place.^44^ We suggest that expression levels of *Itgb* and *Itga* genes vary among myogenic cells or cell lines of different origins and under different culture conditions. The variation in their collective expression repertoire likely results in different experimental outcomes. In addition, the self‐seeding ECM composition on the plastic dish by C2C12 most likely differs from the Matrigel‐coated plastic surfaces for culturing primary myoblasts in our study. As such, the potentially differing ligand(s) available to be used by *Itgb3* in these two paradigms may also impact the outcome. These ligand and receptor differences revealed a prominent role for *Itgb3* in the study by,^30^ but such a role was not observed in this study. Regardless of the difference in these in vitro assays, our in vivo data did not reveal a lineage autonomous role of *Itgb3* in myogenic differentiation.

By contrast, *Itgb1* has been shown to be indispensable for regenerative myogenesis using *Pax7*‐*CreERT2*‐mediated iKO. Here, we reported that *Itgb1* displayed a haploid‐insufficient phenotype, with smaller and fewer regenerated myofibers. Reduction in both of these measurements can be attributed to its level being critical to support robust proliferation of MuSCs. Although regenerated fibers do exist, we did not directly assess differentiation in more detail. Given that both β1‐integrin and β3‐integrin (when partnered with αV‐integrin) can bind fibronectin,^36^ and that fibronectin is critical for MuSC function,^24^ we were surprised to not to find a discernible role for *Itgb3* in the *Itgb1* haploid genetic background. Nevertheless, the *Itgb1* haploid background can be useful to uncover the role of other *Itgb*s in future studies. Similarly, the *Itgb3* cKO and/or iKO model can be used to probe for a compensatory mechanism, if any exist, by other *Itgb* family member(s). Most importantly, our in vivo genetic data clarify the nonessential role of *Itgb3* in the myogenic lineage.

## AUTHOR CONTRIBUTIONS

Chen‐Ming Fan conceived the original idea and wrote a grant to support the project. Andrew Jacob obtained a preliminary set of data. Chen‐Ming Fan and Nathalie Gerassimov designed and performed most of the subsequent experiments and data analyses. Colt Crain and Colin Bilyeu performed a subset of experiments and data analyses, and made the figures. All authors contributed to manuscript writing.

## FUNDING INFORMATION

This work is supported by NIH grants (AR071976 and AR0762644) to CMF, as well as the Carnegie internal fund. Data were generated through accessing research infrastructure at Carnegie Institution for Science.

## DISCLOSURES

The authors declare no conflicts of interests regarding this study.

## ETHICAL STATEMENT

All experiments using mice were conducted in compliance with the protocols approved by the Institutional Animal Care and Use Committee (IACUC) of the Carnegie Institution for Science, and in compliance with the guideline of Office of Laboratory Animal Welfare (OLAW) under assurance number A3861‐01.

## Data Availability

All data are available in the manuscript and upon request.
